# Neonatal Hypoglycemia: A Systematic Review of International and Local Clinical Guidelines with Clinical Implications

**DOI:** 10.3390/jcm15103921

**Published:** 2026-05-19

**Authors:** Camelia Rusu, Melinda Matyas, Boris W. Kramer, Florica Ramona Dorobanțu, Alin Bodog

**Affiliations:** 1 Department of Medical Disciplines, University of Oradea, 410073 Oradea, Romania; 2 Doctoral School of Biomedical Sciences, University of Oradea, 410087 Oradea, Romania; 3 Neonatology Department, “Iuliu Hațieganu” University of Medicine and Pharmacy, 400012 Cluj-Napoca, Romania; 4 Department of Neonatology, Poznan University of Medical Sciences, Fredry St. 10, 61-701 Poznań, Poland; 5 Department of Surgical Disciplines, University of Oradea, 410073 Oradea, Romania

**Keywords:** neonatal hypoglycemia, newborn, screening, operational thresholds, NICU admission, 40% dextrose gel, clinical guidelines, risk factors, persistent hypoglycemia

## Abstract

**Background/Objectives**: Neonatal hypoglycemia is one of the most common metabolic disturbances in the first 24–72 h of life. Despite its frequency, international and local guidelines differ regarding screening strategies, operational thresholds, and escalation pathways. This study is a systematic comparative review of international and local clinical guidelines regarding neonatal hypoglycemia management. **Methods**: We performed a systematic review of major guideline frameworks (AAP, BAPM, PES, CPS, Te Tohu Waihonga New Zealand, and Australian state-based guidance) and compared recommendations for risk factors, screening, treatment thresholds, and NICU admission. **Results**: All guidelines recommended targeted screening of at-risk neonates and immediate treatment of symptomatic hypoglycemia. However, there was no universal agreement on timing of the first blood glucose (BG) measurement, screening frequency, or minimum blood glucose threshold for initiating therapy during the transitional period. Contemporary guidelines endorse 40% oral dextrose gel as first-line therapy in selected cases. **Conclusions**: Despite numerical differences, guidelines converge on core management principles. Further comparative studies are required to define standardized intervention thresholds and optimal measurement strategies.

## 1. Introduction

Neonatal hypoglycemia represents one of the most frequent metabolic disturbances encountered during the early neonatal period, particularly among at-risk newborns such as preterm infants, small-for-gestational-age (SGA) neonates, infants of diabetic mothers, and critically ill neonates. The incidence varies depending on the population studied and the operational threshold applied, but transient low blood glucose concentrations occur in a substantial proportion of healthy newborns during the first hours after birth.

Physiologically, the transition from continuous maternal glucose supply in utero to intermittent enteral feeding after birth requires rapid metabolic adaptation involving glycogenolysis, gluconeogenesis, lipolysis, and ketogenesis. Failure of these adaptive mechanisms, excessive insulin secretion, inadequate substrate availability, or increased metabolic demand may result in clinically significant hypoglycemia.

Although transient neonatal hypoglycemia is often self-limited, prolonged or recurrent episodes have been associated with adverse neurodevelopmental outcomes, including seizures, cognitive impairment, visual dysfunction, and executive dysfunction [1].

Nevertheless, the precise blood glucose concentration associated with neurological injury remains controversial, and no universally accepted diagnostic threshold exists.

International scientific societies have therefore adopted “operational thresholds” intended to guide clinical intervention rather than define a strict biochemical diagnosis. However, substantial variability persists between guideline frameworks regarding screening indications, timing of blood glucose measurements, treatment thresholds, escalation strategies, and criteria for NICU admission.

Given these inconsistencies, a comparative review of current international and local recommendations is clinically relevant. The objective of this systematic review was to compare major neonatal hypoglycemia guideline frameworks regarding screening protocols, operational thresholds, treatment strategies, and recommendations for persistent hypoglycemia, while identifying convergent principles and areas of ongoing controversy.

## 2. Historical Overview of Diagnosis and Treatment

Neonatal hypoglycemia became widely recognized as a clinical entity in the mid-20th century with the development of biochemical glucose assays. Early reports linked low neonatal glucose levels to severe neurological manifestations, including seizures and brain injury. Bedside glucometers introduced in the 1980s enabled rapid detection and monitoring, supporting the distinction between transitional and persistent hypoglycemia. Management evolved from delayed intravenous glucose administration to preventive, stepwise strategies emphasizing early feeding, risk-based screening, and rapid escalation for severe or symptomatic cases. In the past two decades, adoption of operational thresholds and incorporation of 40% oral dextrose gel have reduced the need for intravenous therapy and NICU admission to selected cases.

## 3. Materials and Methods

This systematic review was conducted in accordance with the Preferred Reporting Items for Systematic Reviews and Meta-Analyses (PRISMA 2020, Supplementary Materials File S1) guidelines. No laboratory reagents, commercial materials, instruments, or specialized software requiring manufacturer specification were used in this systematic review.

### 3.1. Search Strategy

A systematic search was performed to identify international and national clinical guidelines on neonatal hypoglycemia. The literature and guideline search was conducted between January and March 2026. Searches included PubMed, Google Scholar, official websites of pediatric and neonatal scientific societies, national health authority publications, and institutional guideline repositories. The search strategy combined the following keywords and Boolean operators: (“neonatal hypoglycemia” OR “newborn hypoglycaemia”) AND (“guideline” OR “recommendation” OR “screening” OR “management” OR “clinical protocol”). Only English-language and Romanian-language documents were included. When multiple versions of the same guidelines existed, the most recent version was selected.

Data synthesis was performed narratively because of the heterogeneity of guideline structures and reported thresholds. The following sources were used: official websites of professional societies and health authorities, including the American Academy of Pediatrics (AAP), British Association of Perinatal Medicine (BAPM), Pediatric Endocrine Society (PES), Canadian Paediatric Society (CPS), Te Tohu Waihonga (New Zealand), and Australian state-based guidance (Queensland Health and Safer Care Victoria), as well as relevant UK and Romanian clinical guidelines (Table 1 and Table 2).

The search covered documents published up to March 2026. Keywords included: “neonatal hypoglycemia”, “guidelines”, “screening”, “blood glucose”, “newborn”, and “management” (Table 3 and Table 4). 

### 3.2. Eligibility Criteria

Inclusion criteria included guidelines that

- Addressed neonatal hypoglycemia management;
- Provided recommendation on screening, diagnosis or treatment;
- Were issued by recognized organization.

Exclusion criteria included

- Non-guideline documents;
- Duplicate/outdated versions;
- Unclear recommendations.

### 3.3. Study Selection

The selection process was performed by two independent reviewers. Titles and full-text documents were screened for eligibility. Discrepancies were resolved by consensus.

### 3.4. Data Extraction

Data extracted from each guideline included

- Target population;
- Screening timing and frequency;
- Operational thresholds;
- Treatment strategies (feeding, oral dextrose gel, or intravenous glucose);
- Criteria for discontinuation of screening;
- Recommendations for persistent hypoglycemia.

### 3.5. Registration

This systematic review was not registered in a prospective database (e.g., PROSPERO).

**Figure 1:**
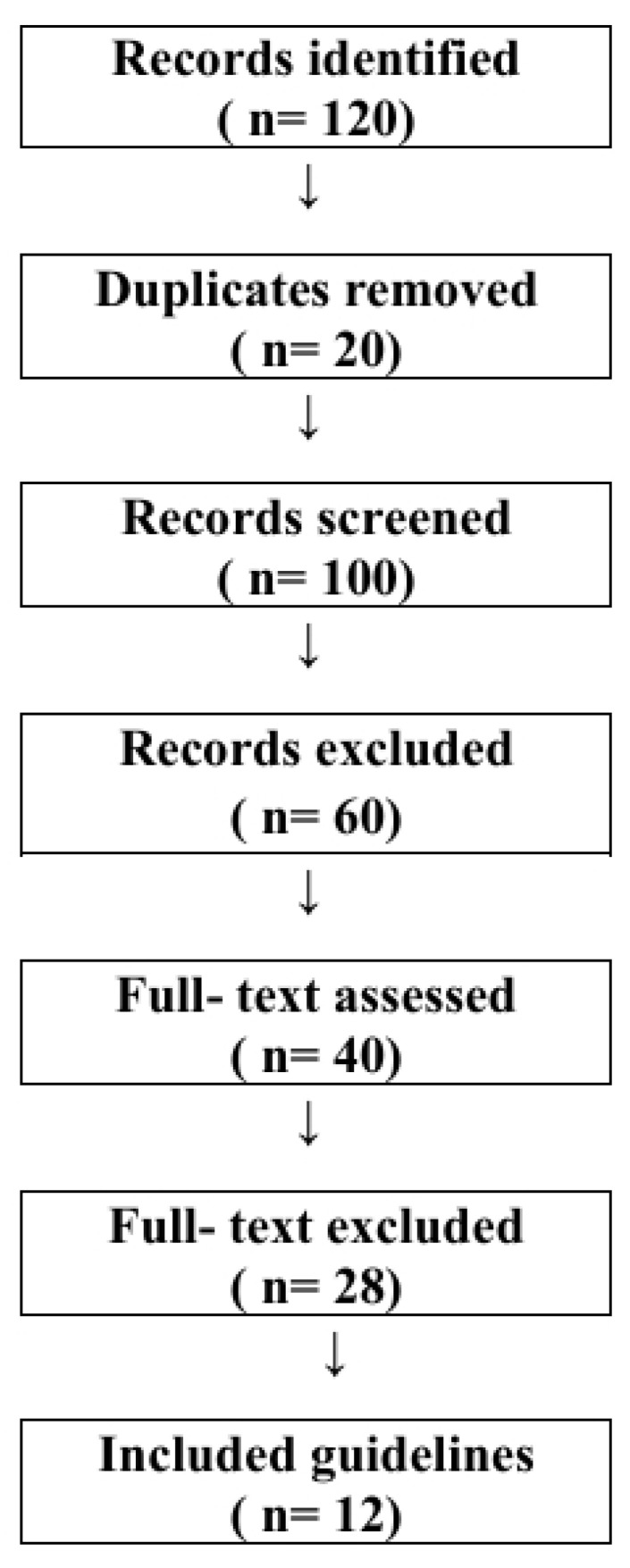
PRISMA 2020 flow diagram of guideline selection process.

The study selection process is illustrated in Figure 1.

## 4. Results

The major international and local neonatal hypoglycemia guideline frameworks included in this review are comparatively summarized in Table 1, Table 2, Table 3, Table 4, Table 5, Table 6, Table 7 and Table 8, highlighting differences in screening strategies, operational thresholds, and therapeutic approaches.

## 5. Integrated Clinical Algorithm for Practice

1. Identification: Symptomatic neonates—measure immediately and treat without delay. Asymptomatic at-risk neonates—screen according to postnatal age and risk factors.
2. Confirmation: Low glucometer values should be confirmed via blood gas analyzer or laboratory testing when diagnostic decisions are required.
3. Transitional Management: Early and frequent feeding; consider 40% oral dextrose gel; re-test at 30–60 min.
4. Escalation: Severe or symptomatic cases require IV glucose infusion and continuous monitoring.
5. Persistent Hypoglycemia (>48–72 h): Obtain critical sample; target higher glucose levels; initiate endocrine–metabolic evaluation (Table 5 and Table 6, Appendix A).

## 6. Discussion

This review highlights substantial convergence across international and local guidance regarding the core principles of neonatal hypoglycemia management, despite variability in numerical thresholds. Across frameworks, early identification of at-risk neonates, prompt intervention for symptomatic hypoglycemia, and avoidance of prolonged or recurrent hypoglycemia remain central objectives. Operational thresholds reflect pragmatic decisions in the context of uncertain outcome-based cut-offs; neurological risk is influenced by severity, duration, recurrence, and individual vulnerability.

The broad endorsement of 40% oral dextrose gel as first-line therapy for selected asymptomatic or mildly symptomatic neonates has major clinical implications: it may reduce the need for intravenous therapy, decrease NICU admissions, preserve mother–infant bonding, and support breastfeeding. These benefits are particularly relevant in settings with limited NICU capacity. Screening strategies remain heterogeneous; therefore, institutions should implement standardized risk-based screening pathways with clear escalation criteria and reassessment intervals (Table 7 and Table 8).

Persistent or recurrent hypoglycemia beyond the transitional period warrants timely etiologic evaluation, including endocrine and metabolic assessment where appropriate. Delayed recognition may postpone diagnosis of conditions such as congenital hyperinsulinism or inborn errors of metabolism.

A further challenge in neonatal hypoglycemia management is the limited accuracy of point-of-care glucometers at low glucose concentrations. False-positive results may prompt unnecessary NICU admission and mother–infant separation, whereas false-negative results may delay treatment of clinically significant hypoglycemia. Accordingly, most contemporary guidelines recommend confirming low readings with laboratory testing or a blood gas analyzer when feasible—without delaying treatment disproportionately.

## 7. Limitations

This review has several limitations. First, the included guidelines differed substantially in structure, terminology, operational thresholds, and year of last update, all aspects limiting direct comparability. Second, some local protocols were institution-based rather than nationally standardized. Third, only English-language and Romanian-language documents were included, potentially excluding relevant recommendations from other regions. Finally, because this review focused on guideline comparison rather than patient-level outcomes, no meta-analysis could be performed.

## 8. Conclusions

There is clear consensus across major clinical guidelines regarding principal risk factors for neonatal hypoglycemia; however, no universal agreement exists concerning the timing of the first postnatal BG measurement in at-risk neonates, the optimal frequency of BG monitoring, or the minimum BG value at which therapy should be initiated during the transitional period. Measurement techniques also vary (portable glucometer, blood gas analyzer, and laboratory confirmation), and each has limitations—particularly at low glucose ranges—which may influence clinical decisions. Further comparative studies are required to define evidence-based intervention thresholds, establish optimal monitoring strategies, identify the most accurate measurement techniques, and clarify long-term neurodevelopmental outcomes. Across guidelines, the overarching objective remains prevention of neuroglycopenia while minimizing unnecessary medicalization and avoidable mother–infant separation.

Nevertheless, important controversies persist regarding optimal intervention thresholds, duration of monitoring, and long-term neurodevelopmental significance of transient low glucose concentrations. Future multicenter comparative studies are needed to establish evidence-based standardized protocols with meaningful outcomes that balance neurological safety with avoidance of unnecessary medicalization.

## Figures and Tables

**Table 1:** Unified table of included neonatal hypoglycemia guideline frameworks.

Organization/Guidelines	Year/Update	Population	Time Window	Key Features	Reference
American Academy of Pediatrics (AAP)	2011	Late-preterm and term infants at risk	0–24 h (core algorithm)	Operational thresholds; feeding first; IV for severe/symptomatic	[2]
British Association of Perinatal Medicine (BAPM)	2017	Full-term infant (birth–72 h)	0–72 h	Integrates 40% dextrose gel; defines severe hypoglycemia	[3]
Pediatric Endocrine Society (PES)	2015	Babies with persistent hypoglycemia/at high-risk	≥48 h + transition	Higher targets after 48 h; evaluation for persistent disorders	[4]
Canadian Paediatric Society (CPS)	2025	At-risk newborns	0–72 h + persistent	Threshold centered on 2.6 mmol/L; gel + feeding; escalation to IV	[5]
Te Tohu Waihonga (New Zealand)	2025	All newborns	Transition + persistent	Detailed algorithm; repeated gel; clear escalation and reassessment	[6]
Australia (Queensland Health; Safer Care Victoria)	2023–2024	At-risk term and preterm infants	0–48 h (transition)	Operational thresholds; gel/feeding; IV escalation; NICU triggers	[7,8]
COLECŢIA GHIDURI CLINICE PENTRU NEONATOLOGIE Ghidul 14	2010	At-risk term and preterm infants	If BG 30–45 mg/dL and clinical sign feed, check BG in 30 min	If BG is still 30–45 mg/dL, IV treatment	[9]
Prevention and Management of Hypoglycaemia (Ashford and St. Peter s Hospitals) UK	2024	All newborns	First feed in 1 h (aim 30 min) BG before 2nd feed	Initiate feed at 3 h; BG pre 3rd feed; if BG > 36 mg/dL, stop BG; recheck	[10]
Hypoglycaemia Neonatal Clinical Guideline (Royal Cornwall Hospitals, UK)	2025	All newborns	2–4 h after birth	IV infusion of 10% glucose at 60 mL/kg/d	[11]

Note: BG = blood glucose; IV = intravenous. Values are reported as presented in original sources; 1 mmol/L ≈ 18 mg/dL.

**Table 2:** Screening strategies for neonatal hypoglycemia (all recommendations preserved).

Guidelines	First BG Check	Routine Screening Frequency	Action if BG 36–45 mg/dL	Action if BG < 36 mg/dL	NICU Admission Threshold	Stopping Screening
SCV	1 h after birth	Pre-feed or every 3 h	Give dextrose buccal gel/EBM/formula; recheck BG in 30 min	Give dextrose gel + feed; recheck in 30 min	BG < 21 mg/dL	After stable values > 47 mg/dL
TWO (NZ)	1–2 h after birth or earlier if symptomatic	Pre-feed	BG > 36 mg/dL: no further monitoring	Pre-feed BG 18–34 mg/dL with clinical signs: oral glucose gel + feed	BG < 19 mg/dL	After 2 normal values
BAPM	Pre-feed before 2nd feed (2–4 h)	Every 3 h	BG 36–45 mg/dL: EBM/formula; recheck before 3rd feed	BG < 36 mg/dL: give dextrose gel; recheck before 3rd feed	BG < 18 mg/dL	BG stable > 36 mg/dL
RCH	Prior to second feed (2–4 h)	Before the 8 h mark	BG > 36 mg/dL and GA > 37 w: stop monitoring	BG < 36 mg/dL: dextrose gel + feed	BG < 19 mg/dL	After stability has been achieved
AAP	Initial feed within 1 h; BG 30 min later	Before each feed (2–3 h)	BG 30–45 mg/dL: feed; recheck in 30 min	BG < 25 mg/dL: IV glucose	BG < 18 mg/dL	Target BG > 45 mg/dL prior to routine feeds
QCG	Before second feed (<3 h of life)	Pre-feed	BG 25–40 mg/dL: refeed/IV as needed	BG < 27 mg/dL: IV treatment	BG < 27 mg/dL	Two pre-feed BG > 36 mg/dL
ASPH/RG	First feed in 1 h (aim 30 min)	Every 2–3 h	BG 27–45 mg/dL: glucose gel + feed	BG < 25 mg/dL: IV glucose	BG < 18 mg/dL	BG stable > 36 mg/dL
RG	NA	If BG 30–45 mg/dL and clinical sign feed, check BG in 30 min	If BG is still 30–45 mg/dL, IV treatment	If BG < 36 mg/dL, IV treatment	If BG 18–29 mg/dL, IV treatment	If BG < 18 mg/dL IV glucose bolus+ IV infusion; recheck in 30 min

Note: EBM = expressed breast milk; GA = gestational age. Content preserved; thresholds as in source protocols.

**Table 3:** Risk factors for neonatal hypoglycemia (original wording preserved).

Category	Risk Factor	SCV	QCG	ASPH	RCH	AAP	TWO	BAPM	RG
Neonatal	perinatal acidosis	NA	NA	NA	YES		YES	YES	NA
Neonatal	preterm infants	YES	YES	YES	YES	YES	YES	YES	YES
Neonatal	low birthweight < 2500 g	YES	YES		YES	YES	YES	NA	NA
Neonatal	SGA	YES	YES		YES	YES	YES	YES	YES
Neonatal	LGA	YES	YES		NA	controversial	YES	NA	YES
Neonatal	clinical signs of hypoglycemia	YES	NA		NA	YES	YES	YES	YES
Neonatal	poor feeding	YES	NA		NA	NA	YES	NA	NA
Neonatal	respiratory distress	YES	YES		NA	NA	YES	NA	NA
Neonatal	temperature instability	YES	NA		NA	NA	YES	YES	NA
Neonatal	birth asphyxia	YES	YES		YES	NA		NA	
Neonatal	sepsis	YES	YES		NA	NA	YES	YES	NA
Neonatal	polycythemia	YES	YES		NA	NA		NA	
Neonatal	post term	NA	YES		NA	NA		NA	
Neonatal	delayed or inadequate feeding	NA	YES	YES	NA	NA		YES	NA
Neonatal	cold stress	NA	YES		NA	NA		NA	
Neonatal	seizures	NA	YES		NA	NA	YES	YES	NA
Neonatal	heart failure	NA	YES		NA	NA		NA	
Neonatal	hyperinsulinism	NA	YES		NA	NA		NA	
Neonatal	inborn errors of metabolism	NA	YES		NA	YES		NA	
Neonatal	syndromes	NA	YES		NA	NA		NA	
Maternal	beta blockers	YES	YES	YES	YES	NA	YES	YES	NA
Maternal	beta agonists	NA	YES		NA	NA		NA	
Maternal	betamethasone after 36 weeks	NA	YES		NA	NA		NA	
Maternal	oral hypoglycemics in the third trimester	NA	YES		NA	NA		NA	
Maternal	maternal diabetes Type I, II, or gestational DM	NA	YES		YES	YES	YES	YES	
Maternal	metformin	NA	NA		NA	YES	NA	NA	
Maternal	insulin	NA	NA		NA	YES	NA	NA	
Maternal	sodium valproate	NA	NA		NA	YES	NA	NA	

Note: YES/not applicable and controversial are reported exactly as in source tables without correction, omission, or reinterpretation.

**Table 4:** Clinical symptoms of neonatal hypoglycemia (all original wording preserved).

System	Symptom	SCV	ASPH	RCH	BAPM	TWO	QCG	AAP	STMH	RG
Neurologic	jitteriness	YES	NA	NA	NA	NA	YES	YES	NA	NA
Neurologic	irritability	YES	NA	NA	NA	YES	NA	NA	NA	NA
Neurologic	high pitched cry	YES	YES	YES	YES	NA	YES	YES	YES	NA
Respiratory	cyanotic episodes	YES	YES	YES	YES	NA	NA	YES	YES	NA
Respiratory	apnea	YES	YES	YES	YES	YES	NA	YES	YES	NA
Neurologic	seizures	YES	YES	YES	YES	YES	YES	YES	YES	NA
Neurologic	lethargy	YES	YES	YES	YES	YES	YES	NA	YES	NA
General	hypothermia	YES	YES	YES	YES	YES	NA	NA	YES	NA
Neurologic	hypotonia	YES	YES	YES	YES	YES	YES	YES	YES	NA
General	altered/poor feeding	YES	YES	NA	YES	YES	YES	YES	YES	NA
Neurologic	altered level consciousness	NA	YES	YES	YES	NA	YES	NA	YES	NA
General	suspected/confirmed infection	NA	YES	YES	YES	YES	NA	YES	NA	NA
Perinatal	perinatal acidosis	NA	NA	NA	YES	NA	NA	NA	NA	NA
Respiratory	respiratory distress	NA	NA	NA	NA	YES	YES	YES	NA	NA
General	sweating/pallor	NA	NA	NA	NA	NA	YES	NA	NA	NA
Neurologic	stupor/coma	NA	NA	NA	NA	NA	YES	YES	NA	NA
Cardiac	bradycardia	NA	NA	NA	NA	NA	YES	NA	NA	NA
Neurologic	eye rolling	NA	NA	NA	NA	NA	NA	NA	YES	NA

Note: YES/NA/YES are reported exactly as in source symptom tables without correction.

**Table 5:** Criteria for admission to NICU in neonatal hypoglycemia (all original wording preserved).

Guidelines	Trigger for NICU Admission	Blood Glucose Threshold	Clinical Signs	Immediate Action	If no IV Access	Reassessment
BAPM	Hypoglycemia and/or clinical signs	BG < 18 mg/dL	±clinical signs	Give IV 10% glucose 2.5 mL/kg	Give glucose gel; feed if it is possible; give Glucagon IM	Recheck BG in 30 min; repeat cycle if BG < 18 mg/dL or clinical signs
QCG	Hypoglycemia and/or clinical signs	BG < 27 mg/dL	clinical signs	Give 10% glucose IV at rate 60 mL/kg/d; BG remains low bolus 1–2 mL/kg	If symptomatic glucose 10% IV 80 mL/kg/d; give glucose gel; give Glucagon IM	Recheck BG in 30 min; recheck in every 30 min after a change in iv glucose
RCH	Hypoglycemia with or without clinical signs	BG < 18 mg/dL or BG < 45 mg/dL with clinical signs	clinical signs	Give 2.5 mL glucose 10%; start IV infusion of 10% glucose at 60 mL/kg/d	Bolus of glucose is NOT necessary	Recheck BG in 30 min; repeat cycle if BG < 18 mg/dL and/or clinical signs
ASPH	Hypoglycemia before the 3rd feed or if symptomatic	BG < 36 mg/dL before the 3rd feed; BG < 18 mg/dL	clinical signs	Give 2.5 mL glucose 10%; start IV infusion of 10% glucose at 60 mL/kg/d	Bolus of glucose is NOT necessary	Recheck BG in 30 min; monitor BG for 24 h
TWO	Severe, recurrent or persistent hypoglycemia	BG < 21 mg/dL; BG < 36 mg/dL after 1 dextrose gel; BG < 18 mg/dL	±clinical signs	Give 0.5 mL/kg dextrose gel; give 2.5 mL glucose 10%; start IV infusion of 10% glucose at 60 mL/kg/d	NO IV ACCESS give Glucagon IM	Recheck BG in 30 min; repeat cycle if BG < 18 mg/dL and/or clinical signs
AAP	Post-feed hypoglycemia	BG < 25 mg/dL (0–4 h); BG < 35 mg/dL (4–24 h)	—	Give 2 mL glucose 10%; start IV infusion of 10% glucose at 80–100 mL/kg/d	—	Recheck BG in 30 min; the goal is BG 40–50 mg/dL
SCV	Hypoglycemia or infant appears unwell	BG < 27 mg/dL; BG < 47 mg/dL after treatment	infant appears unwell	Give 2 mL glucose 10%; start IV infusion of 10% glucose at 60 mL/kg/d	Increase feeding at 80 mL/kg/d OR give IV glucose 10%	Recheck BG in 30 min; recheck BG in 1 h
RG	Hypoglycemia or Baby unwell	BG 18–29 mg/dL; BG < 18 mg/dL	baby unwell	Give 10% glucose IV at rate 60 mL/kg/d; give 2 mL glucose 10%	NO IV ACCESS give Glucagon IM	Repeat cycle until BG > 46 mg/dL; Recheck BG in 30 min

Note: Wording, thresholds, and instructions are reported verbatim from source NICU admission tables.

**Table 6:** Comparative Table of Recommended Thresholds (selected).

Context	AAP (mg/dL)	CPS (mmol/L)	BAPM (mmol/L)	PES (mg/dL)
Asymptomatic ‘at risk’ 0–4 h	IV if <25; re-feed + retest	<2.6	General framework	Not primary focus
Asymptomatic ‘at risk’ 4–24 h	IV if <35; target >45	<2.6 depending on risk	1.0–1.9 gel use	Not primary focus
Severe/symptomatic	Immediate IV + confirm	Urgent treatment ≥2.6	<1.0 emergency	Maintain targets
After 48 h (persistent)	Evaluate if persistent	Higher targets	Investigate if persistent	>60 mg/dL (>48 h); >70 mg/dL disorders

**Table 7:** Convergence of Core Management Principles

Element	AAP	BAPM	CPS/NZ	Australia	Local UK
Targeted screening	Yes	Yes	Yes	Yes	Yes
Immediate IV if symptomatic	Yes	Yes	Yes	Yes	Yes
Use of 40% gel	Optional	Yes	Yes	Yes	Yes
Higher targets > 48 h	Not detailed	Specialist referral	Investigate	NICU referral	NICU referral

**Table 8:** Integrated Characteristics and Key Recommendations

Guidelines	Country	Population	Age Window	Intervention Threshold	Key Interventions
AAP	USA	Late-preterm and term	0–24 h	<25/<35 mg/dL	Feeding; IV glucose
BAPM	UK	Term newborns	0–72 h	1.0–1.9 mmol/L	40% gel; IV
CPS/NZ	Canada/NZ	At-risk neonates	0–72 h	<2.6 mmol/L	Gel + feeding; IV
Australia	Australia	0–48 h newborns	0–48 h	<27 mg/dL	Gel; IV; NICU
UK	UK	All newborns	0–72 h	<18–36 mg/dL	Bolus + infusion

## Data Availability

No new data were created or analyzed in this study. Data sharing is not applicable to this article.

## Supplementary Materials

The following supporting information can be downloaded at: https://www.mdpi.com/article/10.3390/jcm15103921/s1, File S1. PRISMA 2020 Checklist. Reference [12] is cited in the Supplementary materials.

## Appendix A. Glucose Unit Conversion

1 mmol/L ≈ 18 mg/dL (mg/dL = mmol/L × 18)

**mmol/L**	**mg/dL (Approx.)**	**Clinical Context**
1.0	18	Severe threshold (BAPM)
2.0	36	Minimum stabilization threshold
2.6	47	Frequent intervention threshold
3.3	60	Post-48 h target
3.9	70	Target in suspected disorders
